# Quantitative analysis of circadian single cell oscillations in response to temperature

**DOI:** 10.1371/journal.pone.0190004

**Published:** 2018-01-02

**Authors:** Ute Abraham, Julia Katharina Schlichting, Achim Kramer, Hanspeter Herzel

**Affiliations:** 1 Laboratory of Chronobiology, Charité-Universitätsmedizin Berlin, Berlin, Germany; 2 Institute for Theoretical Biology, Humboldt-University, Berlin, Germany; Morehouse School of Medicine, UNITED STATES

## Abstract

Body temperature rhythms synchronize circadian oscillations in different tissues, depending on the degree of cellular coupling: the responsiveness to temperature is higher when single circadian oscillators are uncoupled. So far, the role of coupling in temperature responsiveness has only been studied in organotypic tissue slices of the central circadian pacemaker, because it has been assumed that peripheral target organs behave like uncoupled multicellular oscillators. Since recent studies indicate that some peripheral tissues may exhibit cellular coupling as well, we asked whether peripheral network dynamics also influence temperature responsiveness. Using a novel technique for long-term, high-resolution bioluminescence imaging of primary cultured cells, exposed to repeated temperature cycles, we were able to quantitatively measure period, phase, and amplitude of central (suprachiasmatic nuclei neuron dispersals) and peripheral (mouse ear fibroblasts) single cell oscillations in response to temperature. Employing temperature cycles of different lengths, and different cell densities, we found that some circadian characteristics appear cell-autonomous, e.g. period responses, while others seem to depend on the quality/degree of cellular communication, e.g. phase relationships, robustness of the oscillation, and amplitude. Overall, our findings indicate a strong dependence on the cell’s ability for intercellular communication, which is not only true for neuronal pacemakers, but, importantly, also for cells in peripheral tissues. Hence, they stress the importance of comparative studies that evaluate the degree of coupling in a given tissue, before it may be used effectively as a target for meaningful circadian manipulation.

## Introduction

Daily physiological and behavioral rhythms in mammals are based on cell-autonomous circadian molecular oscillations that are synchronized (entrained) to the 24-h environment by tightly coupled pacemaker cells within the hypothalamic suprachiasmatic nuclei (SCN) [1]. When the SCN are absent, or when peripheral circadian oscillators are cultured *ex vivo*, cell-autonomous circadian oscillations persist; however, due to the lack of a synchronizing signal from the master pacemaker, single cell phases slowly desynchronize from each other, resulting in a significant damping of the population level oscillation [2–7]. The nature(s) of the synchronizing signal(s) of the SCN is (are) still under debate, but so far humoral, synaptic and/or temperature signals have been shown to systemically orchestrate circadian function (for a recent review see [8]). The mechanisms by which these signals might synchronize peripheral oscillations have exclusively been studied in tissues and, hence, on the population level, perhaps involving a certain degree of circadian network dynamics. Quantitative data on single cells, on the other hand, are sparse. In light of the ongoing discussion about whether peripheral circadian oscillators can or do couple to each other [9;10], or whether they are just an accumulation of independent cellular oscillators [11], we asked if synchronizing signals act differently on single cells compared to peripheral tissues. Since models predict that the extent of zeitgeber effects are larger in uncoupled systems [12–15], we want to know whether results of external zeitgeber action are similar, independent of whether they act on tissues or single cells. In order to answer this question, we used a novel technique to accurately trace and quantitatively analyze single cell responses to temperature signals using long-term bioluminescence measurements. PER2::LUC mouse primary fibroblasts and SCN neurons served as models for mammalian peripheral and central oscillators. Temperature was selected as external stimulus, because temperature fluctuations within the physiological range have been shown to be effective as a synchronizing stimulus on tissues as well as single cells, including fibroblasts [12;16–20]. Furthermore, it is a non-invasive technique (as compared to mechanical or chemical perturbations). Exposing cells to external temperature cycles which resembled physiological body temperature fluctuations, and measuring their responses in real-time before, during, and after the signal for up to 14 days, we extracted single cell circadian characteristics, like period, phase and amplitude responses, and compared them to circadian parameters at the tissue and organismal level. We found that single cells respond to external stimuli as multicellular oscillatory systems do, adjusting their intrinsic periods and phases according to the external temperature cycle, and showing similar circadian responses to single stimuli. However, we also identified differences: the phase relationship between single cell circadian oscillations and the stimulus is–unlike in our own results from multicellular systems (primary lung tissues, [21])–not dependent on the intrinsic period. This fundamental difference suggests that circadian phase relationships, which constitute a biologically relevant characteristic of environmental circadian entrainment [22], critically depend on circadian network dynamics rather than on cell-autonomous circadian properties. In addition to our own findings, a study on fibroblast culture densities highlighting the fact that network dynamics might as well play a role in peripheral oscillators [23], also supports this conclusion. For central oscillators within organotypic slices, the impact of intercellular coupling on circadian temperature responsiveness had already been shown [12;16;24]. Considering that coupling in these studies refers to 3-dimensional organotypic networks only, we aimed to study 2-dimensional coupling strategies by investigating different pacemaker densities, which represent different degrees of intercellular communication [25]. We found that less densely cultured circadian oscillators display a much higher responsiveness to physiological temperature fluctuations, suggesting once more that circadian network dynamics by means of intercellular communication, rather than the properties of the individual oscillators, essentially determine how a system responds to an external signal.

## Material and methods

Procedures were authorized by and performed in accordance with guidelines and regulations of the German animal protection law (DeutschesTierschutzgesetz).

### Cell culture of primary mouse ear fibroblasts

3-7-month old PER2::LUC mice [26] and 2–3 months old C57Bl/6 J mice, bred and raised in our animal facility (FEM, Charité, Berlin) were killed by cervical dislocation, their outer ears sectioned into very small pieces, and transferred to Dulbecco’s Modified Eagle Medium (DMEM, Gibco, Life technologies, Germany), supplemented with 20% FBS, 100u/100u penicillin/streptomycin (Gibco), and 2.5μg/ml amphotericine B (referred to as CM20+). Subsequently, the tissue suspensions were supplemented with 200μl of Liberase TM Research Grade (Roche, Germany) and incubated overnight at 37°C/5%CO_2_. Following incubation, the dispersed tissues were centrifuged, washed, seeded in CM20+ in appropriate culture dishes, and maintained at 37°C/5%CO_2_. After a minimum of six days and zero cell passages, and a maximum of 22 days and two cell passages, PER2::LUC and C57Bl/6J fibroblasts were mixed at about 1:20, and seeded at high density in 35mm glass bottom dishes with a 10mm glass diameter (MatTek, USA) in CM20 (= CM20+ minus amphotericin B). At the day of imaging cells were confluent. To prevent proliferation of fibroblasts upon recording, the serum content in the recording medium was lowered to 3%. The recording medium was adjusted to a normal gaseous environment (air) as described in (Abe *et al*. 2002) and supplemented with 0.18mM beetle D-luciferin (BioThema, Sweden).

### SCN dispersal culture

300μm SCN slices from 4-7-day old PER2::LUC mice were obtained as described in (Abraham *et al*. 2010) and collected in chilled Hank’s balanced salt solution (HBSS, pH 7.2, Sigma), supplemented with 1mM kynurenate and 0.05mM DL-APV (both Sigma). The bilateral SCN were punched out using a 22-gauge neuro punch (Fine Science Tools, Germany), the punches of 9–10 pups were pooled and dispersed by using papain (Aton *et al*. 2006). Approximately 3000 cells/mm^2^ (dense) and 600 cells/mm^2^ (medium dense) were cultured on 1.8 mm^2^ in a poly-D-lysine/laminin-coated MatTek dish (MatTek Corporation, USA) and maintained in 3ml of DMEM, supplemented with B27 (Invitrogen) and 10% FCS. After 7 days *in vitro*, the culture medium was supplemented with 20μM of cytosine arabinoside (Ara-C, Sigma) to control glia proliferation. Bioluminescence imaging of SCN dispersal cultures was performed in air-adjusted culture medium supplemented with Ara-C and 0.18mM luciferin after 9 days *in vitro* at the earliest. During imaging the final densities of bioluminescent neurons were determined to be **≈**220 and **≈**40 neurons/mm^2^ for the dense and medium dense cultures, respectively.

### Bioluminescence imaging

Imaging was performed with an inverse setup in a light-tight chamber using a 10x objective (Zeiss FLUAR, 10x, N.A.: 0.50, Germany) connected by a straight tube with an intensified CCD camera (XR/Mega-10Z 30S, Stanford Photonics, USA). Culture dishes were sealed with grease and placed on the imaging stage under transparent glass heaters, attached to a temperature controller (TCII, Cell Micro Controls, USA) that, by means of a temperature feedback, kept the cell culture at a constant 37°C. Images were obtained at a camera temperature of -20°C with a gain setting of 1560 and an exposure time of 30min over the course of at least 10 days. Using a custom-build LabVIEW (National Instruments, USA)-based software (Raik Paulat, Medizinisch-Technische Labore, Charité-Universitätsmedizin, Berlin), the temperature controller was programmed to run six repeats of either a T20 (10h of 33°C and 10h of 37°C), or a T24 (12h of 33°C and 12h of 37°C) temperature cycle with a temperature difference of 4°C. The temperature cycles started at about 2–4 days after the start of the imaging, and ended about 3–4 days before the end of the recording.

For the comparison of dense and medium dense SCN dispersal cultures, only T20 temperature cycles with a difference of 2°C were used.

### Analysis of time-series data

#### a) Tracking of single cell bioluminescence

Image processing was performed with Fiji [27]: First, the raw image sequences were filtered using the Kalman Stack Filter plugin (www.fiji.sc/Kalman_Stack_Filter). Subsequently, in order to facilitate tracking of single cells images were automatically adjusted for brightness and contrast and Kalman-filtered again. Brightness adjustment served visual purposes only and did not alter measurement values. Outliers were removed from images using the built-in -Fiji option with the settings "rad 1" and "threshold 100". For fibroblast movies, single cell bioluminescence was traced using the SpotTracker plugin [28], which enabled us to accurately track cells that move and oscillate at the same time. We then calculated the signal strength as the mean over a small number of pixels surrounding the traced pixel with the programming language R. Neurons in SCN dispersal cultures were almost immobile. Therefore, tracing of single neuron bioluminescence was done manually by drawing a region of interest (ROI) around a single neuron and measuring the mean gray level using Fiji.

Cell viability was verified throughout the experiments by tracing population level PER2::LUC bioluminescence levels. Cultures that exhibited a strong reduction in bioluminescence or a permanent loss of rhythmic bioluminescent activity were excluded from the analysis.

#### b) Data processing

Maximal measurements of single fibroblast bioluminescence were normalized to gray level 255 to ensure comparability across movies.

Both, neuronal and fibroblast measurements were background-subtracted and trend-eliminated. The mean background noise for each image was calculated as the mean of four different ROIs that clearly did not contain any cells. For trend elimination, we subtracted a 24h-running average from the data. In order to ensure comparability of magnitudes, we added the average minimal gray level to the trend-eliminated data.

#### c) Determination of circadian parameters

In order to determine amplitudes and periods, we fitted the cosine function

$$f(t)=a\cdot cos\left(\frac{2\pi }{\tau }\cdot t-\theta \right)+offset$$

to the processed data in the time ranges *Before*, *During* and *After* the temperature cycles. *a* = amplitude, *τ* = period, whereas parameters *θ* and *offset* shift the function along the axes. We estimated the initial values for the iterative non-linear least square fit with a fast Fourier transform. For period distributions, only the last 3 temperature cycles were considered for the *During* condition in order to minimize the effect of transients. Only periods between 18h and 30h were considered “circadian” and included in the analysis. Phases (peak times) were determined with a peak picking procedure: processed data were smoothed with an 11h-running average, and a quadratic parabola fitted within a range of about 30 data points around the potential peaks. The extrema of the parabola were defined as peak times. Relative amplitudes were calculated from the absolute amplitudes divided by the mean of the unprocessed data.

#### d) Calculation of circadian phases

Peak times were determined as described in c) with the time axis rescaled to the respective experimental phase. For *Before* and *After*, circadian phases were determined in circadian hours (τ *Before* /24h), and for *During* the time axis of one circadian day (= 24 circadian hours) was rescaled to 20h for T20, and to 24h for T24. Hence, circadian time (CT) 12 refers to the middle of the circadian day and the end of the cold phase, independent of T cycle length.

#### e) Calculation of phase shifts

Phase shifts in response to the first 10-h cold phase (from here on referred to as cold stimulus) were calculated as follows: the *Before* data was extrapolated using a cosine fit to define the expected peak. A quadratic parabola was then fitted to 30 data points surrounding the measured peak upon the first cold stimulation. Its extrema defined the actual fitted peak. The phase shift in response to the cold stimulus is the difference between the expected and the actually fitted peak in circadian hours (= hours x τ/24). Negative values denote phase delays, while positive values represent phase advances. The phase of the onset of the stimulus was determined in reference to the maximum expression of PER2 in peripheral tissues (= CT18) (Yoo *et al*. 2004).

Statistical analyses were performed using “R” version 3.2.2 (2015-08-14), and “Oriana 3” (Kovach Computing Services, UK).

## Results

### Temperature cycles affect single cell circadian oscillations depending on the relationship between internal and external period

**Fig 1:**
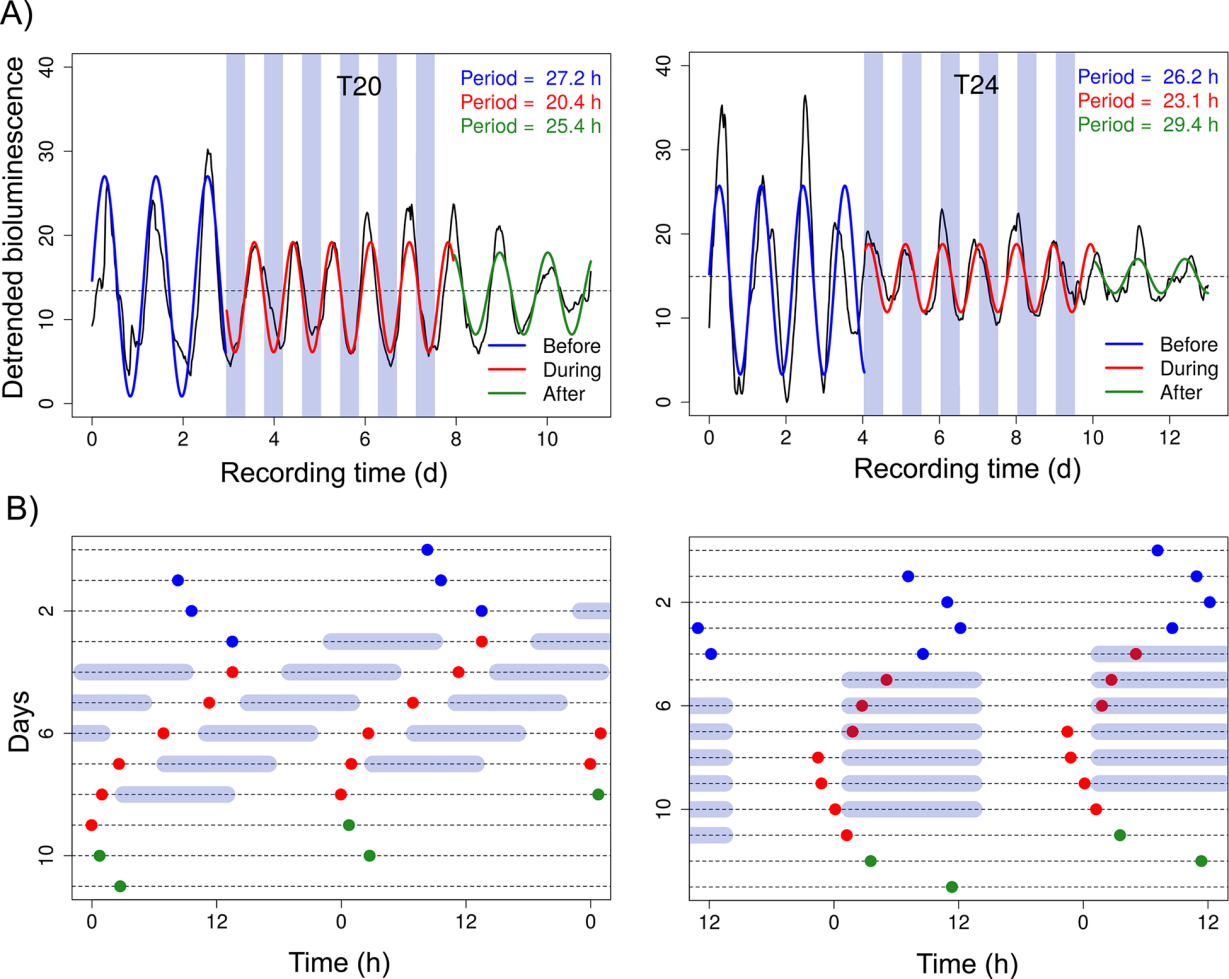
Circadian single cell bioluminescence of primary mouse fibroblasts follows external temperature cycles. A: Representative examples of PER2::LUC bioluminescence (gray values) emitted from single mouse ear fibroblasts in confluent cell cultures exposed to six repeats of 10h of 33°C (cold): 10h 37°C (warm) (T20, left) or 12h cold: 12h warm (T24, right), respectively. The periods of the oscillations were determined by cosine fit *Before* (blue), *During* (red), and *After* (green) the temperature cycles. Dotted horizontal line: mean magnitude of bioluminescence. B: Double-plotted peaks (dots) of the cells shown in A) reveal that oscillations assume a semi-stable phase relationship with the cold phase. Light blue: cold phase.

**Fig 2:**
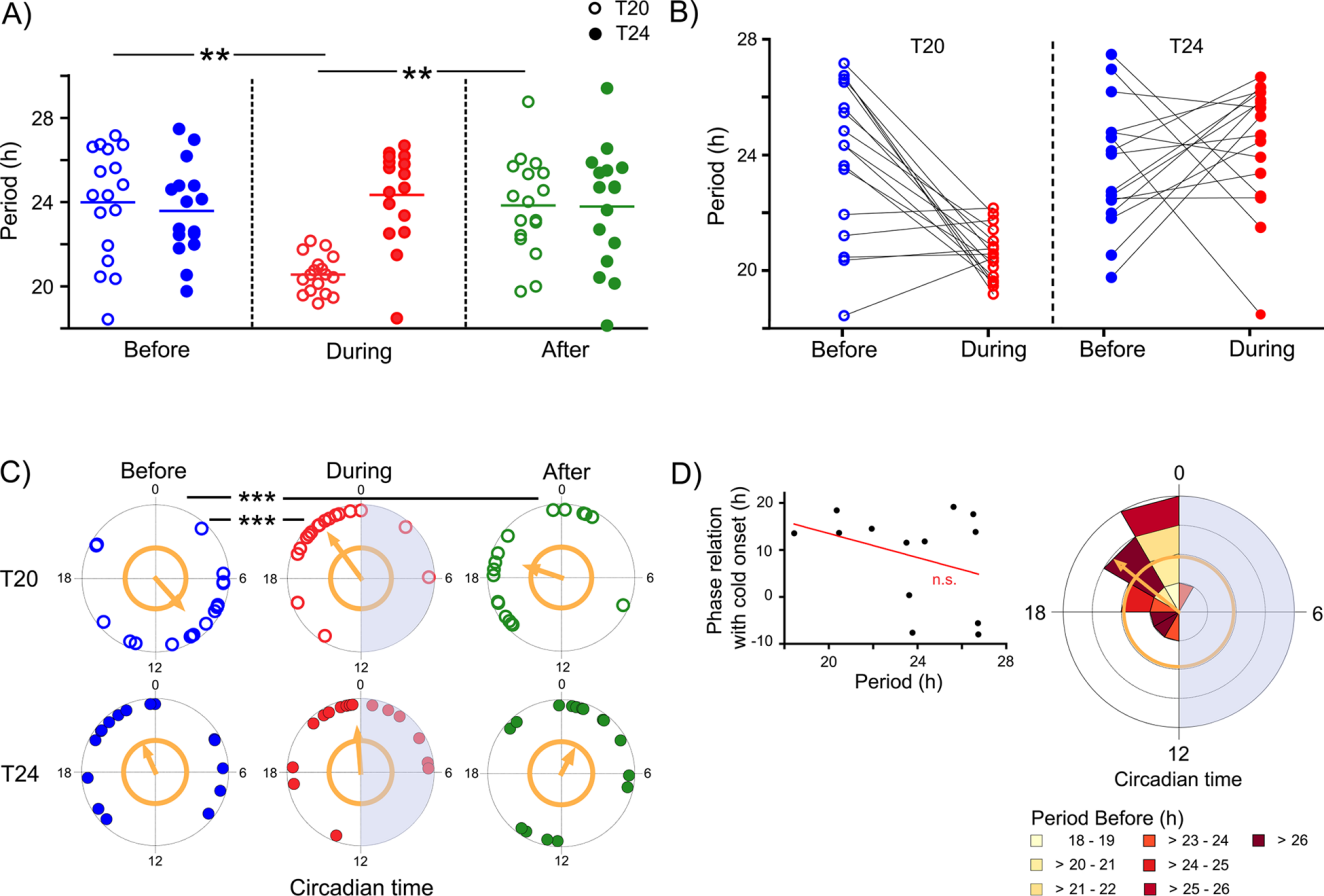
Temperature cycles affect single fibroblast periods and phase relationships. A: Scatterplot showing the distribution of periods displayed by single cells exposed to T20 (open circles) and T24 (closed circles) temperature cycles *Before*, *During*, and *After* the temperature paradigm. Each dot represents a period measurement of a single cell; the mean is marked by a horizontal line. Only cells that display circadian periods in all three conditions were included in the analysis. A two-way repeated measures ANOVA revealed that both treatment (T-cycle) and time (phases *Before*, *During*, and *After*,) and their interaction have significant effects on average periods (p = 0.013, p = 0.03, and p = 0.0008, respectively). Mean periods of cells exposed to T20 are significantly different between *During* and *Before*/*After* the temperature cycles (Two-Way-ANOVA for repeated measures, Bonferroni posttests, ** = p<0.001, n = 17), while periods in T24 do not change in response to temperature (Two-Way-ANOVA for repeated measures, Bonferroni posttests, p>0.05, n = 16). B: Individual period developments of single cells in constant temperature conditions (*Before*) and *During* temperature cycles in T20 (open circles) and T24 (closed circles) conditions. Each dot represents a period measurement of a single cell; repeated measures are connected by a black line. The plot shows that the broad period distribution *During* T24 is not a result of cellular periods being insensitive to the temperature paradigm, but rather due to very variable individual period responses. This is in contrast to T20 where the majority of cells respond with period shortening. C: Polar plots depicting single cell PER2 circadian peak phases *Before* (last phase before temperature cycles), *During* (mean of the last two phases during), and *After* (first phase after) the temperature cycles for T20 (open circles, n = 17) and T24 (closed circles, n = 16). Each dot represents the peak phase of a single cell measured in the respective condition. The direction of the orange arrow denotes the mean phase. The phase population clusters significantly when the arrow crosses the corresponding circle (Rayleigh's uniformity test, p<0.05). The blue shaded areas of the polar plots *During* denote the cold phases. Mean circadian phases are significantly different between *Before* and *During*/*After* T20 (Watson-Williams-F-Test, *** = p<0.0001). D: Left: The relationship of the PER2 peak phase with the onset of the cold phase (= mean of the phase relationships with the last two cold phases) is not significantly dependent on the single cell's intrinsic period (τ *Before*) (Linear regression, r^2^ = 0.08, p>0.05, n = 17). Only cells subjected to T20 temperature cycles were included in the analysis, provided that they exhibited a τ *During* that was close to (+/-1h) the external temperature cycle. Right: two-variable polar plot showing the frequency distribution of PER2 peak phases depending on the intrinsic period (τ *Before*) of the cells plotted in the left panel of D (n = 13). The radius of a wedge (concentric intervals: 2) represents the number of cells displaying a circadian peak phase within a 2h-bin (for a description of circadian phase determination see p.7, section d)), while color-coded segments denote the intrinsic periods (τ *Before*). There is no clear relationship between intrinsic period and phase, which supports preliminary conclusions from our linear analysis to the left.

Primary mouse ear fibroblasts, cultured at confluency and passaged (trypsinized) one to three times after explantation, displayed circadian PER2-driven bioluminescence before, during, and after exposure to 6 cycles of either a 10-h warm/10-h cold (T20, Fig 1A, left, S1 Movie), or a 12-h warm/12-h cold (T24, Fig 1A, right, S2 Movie) temperature cycle. Bioluminescence peaks of the two representative cells shown in Fig 1A show that initially oscillations free-run with a period slightly greater than 24h (Fig 1B, blue dots), while fibroblast periods clearly shorten upon exposure to temperature cycles—depending on the period of the external stimulus—with peaks approaching an almost stable phase relationship with the cold phase (Fig 1B, red dots). Subsequently, upon release into constant temperature conditions, bioluminescence appears to free-run again with periods similar to the initial ones (Fig 1B, green dots). To test whether these individual observations follow a general principle, we performed a systematic analysis of the periods in all three conditions: while mean periods before the temperature cycles were close to 24h in all cells, periods significantly shortened to almost 20h in cells exposed to a T20-cycle (Two-Way-ANOVA for repeated measures, Bonferroni posttest, ** = p<0.001, n = 17), but period distribution remained unaltered in cells exposed to a T24-cycle (Fig 2A, S1 Movie and S2 Movie). As expected, following the release into constant temperature, all cells displayed periods similar to their initial periods again, resulting in a significant lengthening for T20-cells (Two-Way-ANOVA for repeated measures, Bonferroni posttest, ** = p<0.001, n = 18), while cells that had been exposed to a T24-cycle showed no significant difference in periods throughout the experiment. It is also noteworthy, that the exposure to an external stimulus with a period that is significantly different from the internal circadian period leads to a noticeable reduction in period variation (Coefficient of variation (CV) T20, *Before*: 11%, *During*: 4.3%), while variability in cells which have been exposed to an external period that is close to the internal period was largely unaltered (CV T24 *Before*: 9.3%, *During*: 9.1%). Nevertheless, individual period developments show that cells do respond to T24 temperature cycles, but that, in contrast to T20, the individual responses are highly variable, so that the overall variability appears unaltered (Fig 2B). A similar effect was observed for phasing (Fig 2C): exposure to T20, a temperature cycle that was about 4h shorter than the average τ *Before* (**≈**24h, see Fig 2A), resulted in a significant phase shift of about 12 circadian hours (Fig 2C, T20 *Before* and T20 *During*, Watson-Williams-F-Test, p<0.0001) and an increase in phase clustering (T20, Rayleigh's uniformity test: Z *Before*: 4.35; Z *During*: 8.17). The phase shift persisted even after the external T-cycle had ended (Fig 2C, T20 *After*) indicating the absence of masking by temperature. In contrast, average phasing was not significantly altered, when internal and external periods were close (Fig 2C, T24). However, the phase concentration slightly increased upon exposure to temperature cycles (T24, Rayleigh's uniformity test: Z *Before*: 1.86; Z *During*: 4.81).

### Phase relationships are independent of the cell’s internal period

Considering the obvious importance of internal vs. external period, we tested whether the internal period also systematically determines the phase relationship with the external stimulus, analyzing the mean phase relationship of peak bioluminescence with the last two temperature cycles depending on the intrinsic period (τ *Before*). Since fibroblast single cell oscillations and, consequently, their phase relationships appear rather noisy (see Fig 1), we were concerned that a potential systematic relationship between internal and external period might be obscured by inherent noise and/or by incomplete entrainment. To overcome this limitation, we excluded cells displaying a τ *During* that deviates from the external temperature cycle by more than one hour. Nonetheless, linear regression was still not significant (Linear regression, R^2^ = 0.08, p>0.05, n = 17, Fig 2D, left), indicating that there is no systematic relationship between intrinsic period and phase in these cells. Considering that the analysis of circadian phases on a linear scale is problematic, we re-plotted the phases on a circular scale and identified the corresponding internal periods (τ *Before*; Fig 2D, right). Again, a simple systematic relationship between internal period and phase could not be detected.

### Single cell phase responses are similar to those of organisms and tissues

**Fig 3:**
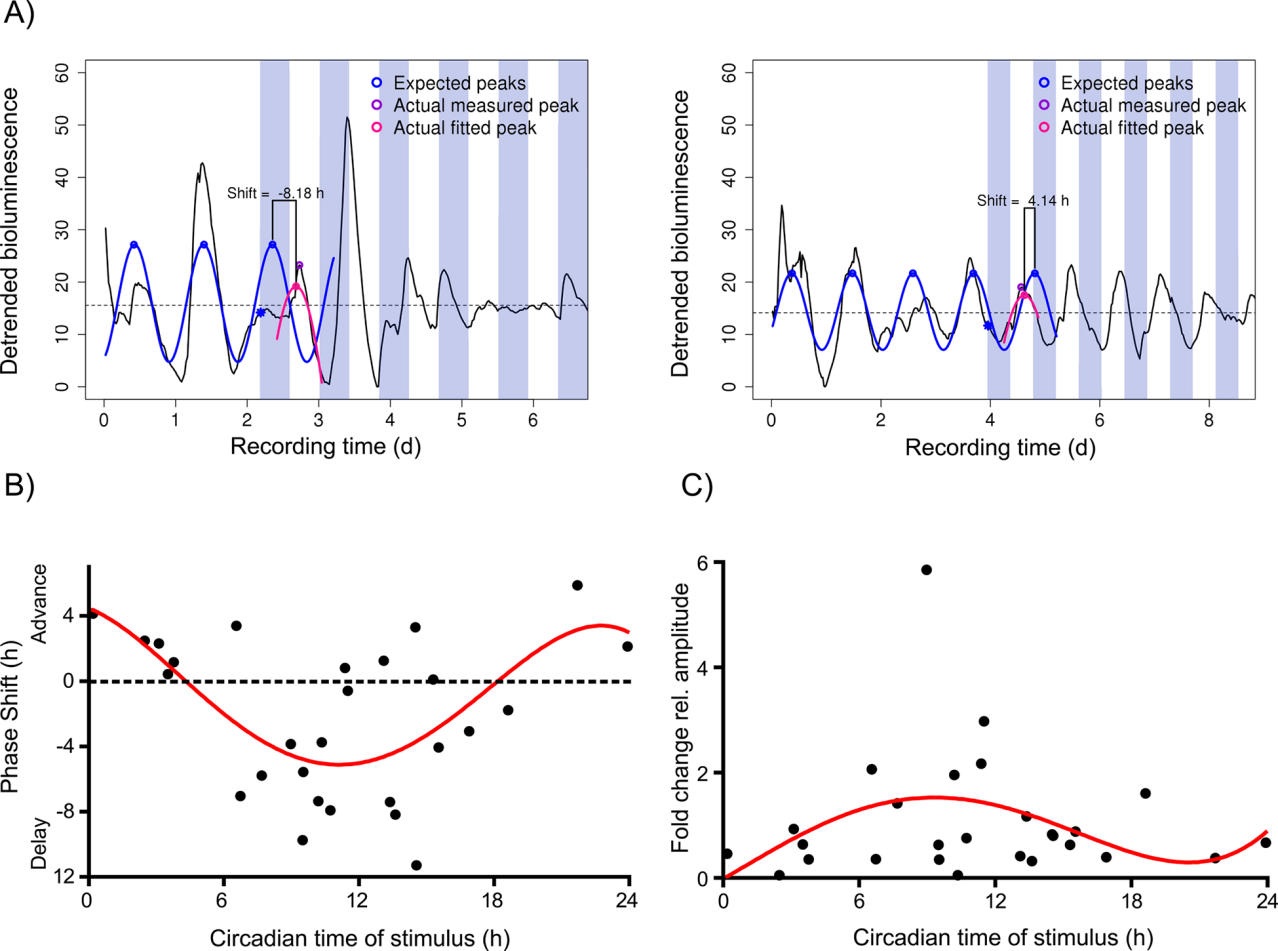
Individual fibroblasts respond to external stimuli roughly as predicted from tissues and whole organisms. A: Representative examples of a phase delay (negative shift, left) and a phase advance (positive shift, right) of single cell PER2::LUC bioluminescence (gray values) in response to a 10h cold phase hitting the oscillation at CT14 (left) and CT0 (right). The peak of PER2::LUC in fibroblasts was defined as CT18, in reference to [26]. Dotted horizontal line: mean magnitude of bioluminescence. B: The phase responses reveal that cells which have been hit by the cold phase between CT6 and CT18 mostly respond with a delay phase shift (mean: -3.9±1.0h SEM, n = 20). At other circadian times (CT 18 to CT6) cells respond with a phase advance (mean: 2.6±0.7h SEM, n = 7). Mean phase shifts within these two circadian ranges differ significantly (t-test, p<0.001). Dots: phase shifts of single cells; line: fourth order polynomial fit. C: The amplitude response curve (ARC) depicts the relative amplitude response (fold change) as a function of the circadian time of the onset of the 10-h temperature pulse. In contrast to the phase responses (B), there is no significant difference in the relative amplitude responses between CT6 to CT18 and CT18 to CT6 (Mann Whitney test, p>0.05, n = 20). Dots: relative amplitude changes of single cells in response to a cold stimulus; line: fourth order polynomial fit.

In order to complete our characterization of single cell oscillator responses, and to potentially uncover further differences with respect to multicellular systems, we investigated the effect of a single temperature event on single cell circadian phasing, aiming to create something similar to a phase response curve (PRC), which is known from multicellular organisms. For this purpose, we analyzed the responses towards the first cold phase only. Acknowledging the fact that temperature stimuli of 10–12 hours are rather long, encompassing a range of circadian times, we excluded the data on 12-h pulses. Since we do not know which part of the temperature stimulus is relevant for the response (onset, offset, duration, delta T), we arbitrarily defined the onset of the cold phase as the circadian time of stimulus. When cold pulses started during the ascending limb of the PER2::LUC oscillation (corresponding to CT14), cells respond with a phase delay (Fig 3A, left), and when hit during the descending limb of the oscillation (corresponding to CT0), they show a phase advance (Fig 3A, right). This was detected for the majority of cells, as shown in Fig 3B: 14/19 cells responded with a phase delay when the cold phase hit between CT6 and CT18 (ascending limb), while 7/8 cells responded with a phase advance when stimulated at other circadian times (descending limb). Correspondingly, mean phase shifts within these two circadian ranges differ significantly (t-test, p<0.001). In contrast, amplitude responses did not show a systematic pattern (Fig 3C).

### Temperature effects on single neurons are determined by cellular connectivity

**Fig 4:**
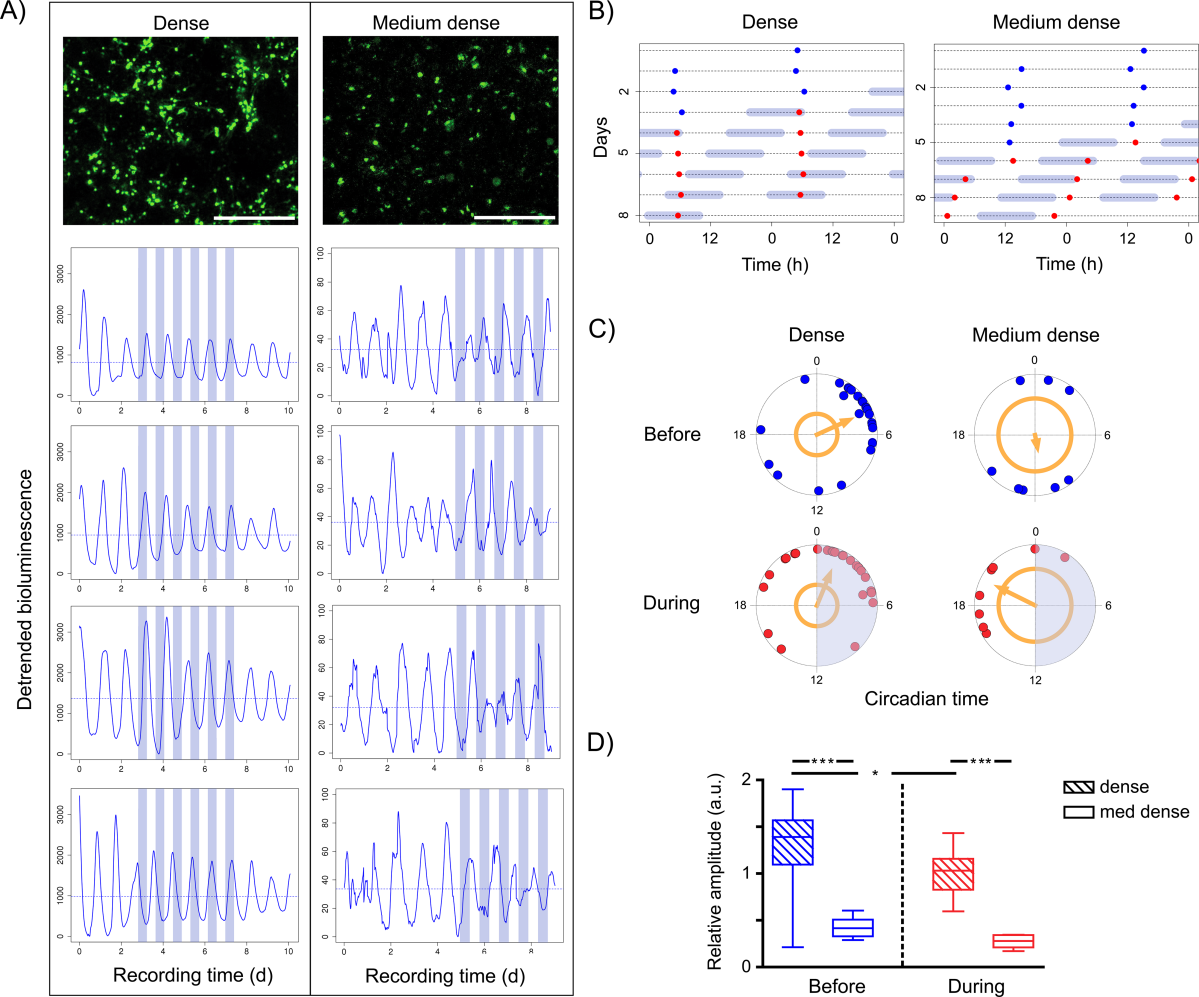
The degree of intercellular communication determines the impact of temperature cycles on single SCN cells. A: PER2::LUC SCN dispersals were cultured at high (**≈**220 neurons/mm^2^, top left) and medium density (**≈**40 neurons/mm^2^, top right), and bioluminescence (gray values) from single cells monitored over the course of several days. Scale bar: 500μm. On day 3 or 5, cells were exposed to a T20 temperature cycle with deltaT = 2°C. Four representative single cell traces of a dense (left panel) and a medium dense culture (right panel) reveal that oscillations appear more robust in dense cultures. Light blue: cold phase (35°C); horizontal line: mean magnitude of bioluminescence. B: Peak bioluminescences (dots) from two representative single neurons in a dense and a medium dense SCN dispersal culture (same cells as top panels in A) depending on the time of day. Single cell SCN oscillations in the dense culture are clearly less perturbed by a temperature cycle (peak bioluminescence "free-runs" through the temperature cycle) compared to the medium dense culture (cells tend to adjust their period to the external cycle). Light blue: cold phase. Data were double plotted for a better visualization. C: Polar plots depicting single neuron PER2 circadian peak phases repeatedly measured *Before* (last peak before temperature cycles) and *During* (fourth peak during temperature cycles) in a dense (n = 25) and a medium dense (n = 8) SCN dispersal culture. Each dot represents the peak phase of a single cell measured in the respective condition. The direction of the orange arrow denotes the mean phase. The phase population clusters significantly when the arrow crosses the corresponding circle (Rayleigh's uniformity test, p<0.05). In the dense culture, peak phases cluster significantly around CT2-4, independent of whether a temperature signal is present or not. In contrast, phasing in the medium dense culture appears rather random *Before*, but shows a phase concentration *During*, with the mean peak phase being pushed to the warm phase. D: The relative amplitudes of single cell oscillations in the dense culture (n = 25) are significantly higher than those in the medium dense culture (n = 8), independent of the external signal (One-Way ANOVA with Tukey's multiple comparison posthoc tests; *** = p<0.0001). Relative amplitudes in a dense culture significantly decrease upon exposure to a temperature signal (* = p<0.01). Displayed are boxplots with min/max.

Considering that phase relations to an external stimulus appear not to depend on the internal period in single cells, but, in contrast, appear to be heavily dominated by the internal period in oscillating tissues and whole organisms, we hypothesized that cellular coupling is critical for regular circadian output. In light of the ongoing debate whether fibroblasts can couple or not, we used SCN neurons, which are well known to establish neural connections in high density dispersal cultures, in order to further investigate the role of intercellular communication for circadian synchronization. Accounting for the fact that central homeothermic tissues usually experience smaller physiological temperature changes than peripheral ones, we reduced the temperature difference to 2°C. Single neurons of high density SCN dispersals display high magnitude, robust circadian oscillations that are completely unperturbed by the external temperature cycle (T20, Fig 4A and 4B, left, S3 Movie). In contrast, SCN neurons in medium density dispersals oscillate with a more than 20-fold reduced magnitude and with less precision (Fig 4A, right column). They are clearly perturbed by the T20 temperature cycle, which is most prominently reflected in phasing: while PER2::LUC rhythms appear synchronized and unaltered by temperature in the dense culture, less densely cultured neurons go from a rather random distribution *Before* to a more clustered distribution at the end of the warm phase (Fig 4C, *During*). Coincidently, there is a significant reduction in rhythm amplitude of less densely cultured neurons (Fig 4D).

## Discussion

Employing long-term bioluminescence imaging of single fibroblasts, simultaneously exposed to repeated temperature cycles, we were able to extract circadian parameters of single cells in response to external signals. These results generate new insights into entrainment mechanisms, because these investigations have as yet only been carried out in tissues which, by definition, constitute a network of multiple oscillators that might or might not communicate with each other. Since temperature cycles have been shown to exert entraining effects on peripheral oscillators [12;16;17], our temperature paradigms which mimic daily temperature fluctuations that typically occur in the periphery of homeotherms [29] represent a biologically relevant signal. Accurately tracing bioluminescence rhythms of dim and motile single cells over a long duration can be challenging, particularly, if pixel array binning is not desired because it might compromise the distinction of individual cell traces. We solved these problems as follows: First, we seeded bioluminescent cells in a low density, while keeping them stationary by confluent non-bioluminescent fibroblasts and by adding a low percentage of serum to the recording medium, interfering with cell division and motility, but not with viability. Second, we developed a novel technique to accurately track long-term, high-resolution clock gene-driven bioluminescence rhythms of single fibroblasts, adapting from a freely available, easy-to-use ImageJ (NIH, USA) plugin. We can exclude that our data were compromised by a direct impact of temperature on the luciferin-luciferase interaction, because we never observed an immediate increase or decrease of the overall bioluminescence level in response to a temperature change.

### Single oscillators behave similarly to tissue level oscillators…

As expected from peripheral tissues (Abraham *et al*. 2010), peripheral single cells respond to temperature cycles by adjusting their intrinsic periods to the extrinsic period, along with a reduction in intercellular period variability (Fig 2A). Period adaptation and reduction of variability were significant only when mean intrinsic and extrinsic periods were different by several hours (4h in our case), while period responses were very variable when intrinsic and extrinsic periods were close (Fig 2A and 2B). Despite being counterintuitive, this phenomenon might be explained by the fact that in T24 the stimulus phase (12 h) incorporates a wider CT range of the phase response curve than in T20 (10h) (compare single cell phase responses in Fig 3B). This way, delays and advances might be more balanced in T24 than in T20, resulting in smaller netto phase shifts of higher variability (32). Also in line with this is an increase in phase clustering upon temperature exposure for T20, but not for T24 (Fig 2C). However, increased clustering, as well as the significant phase shift for T20, might also be brought about by the majority of T20 cells being hit by the first cold between CT6 and CT18 (Fig 2C, T20 *Before*). This is a phase range, in which these fibroblasts respond stronger to temperature pulses than at other times (Fig 3C). In contrast, the majority of the T24 cells were in opposite circadian phase *Before* (Fig 2C, T24), and, hence, likely were hit by the first cold phase between CT18 and CT0, a phase range, where single cells have been shown to be less phase responsive to temperature (Fig 3C).

Other characteristics of circadian entrainment, the establishment of a stable phase relationship with the zeitgeber and the temporal preservation of the entrained phase upon removal of the entraining signal, however, were not clearly evident in our single cells (Figs 1B and 2C). Consequently, we cannot be certain whether our cells were entrained by the temperature paradigm or not. The results suggest that the majority of cells were partially entrained. Entrainment theory and experimental findings predict that a longer exposure to temperature cycles may result in complete and stable entrainment [30]. However, this is mere speculation, since the noise (see Fig 1) inherent to fibroblast single cell oscillations makes accurate phase determination difficult.

### … but some entrainment parameters seem to depend on network properties

In contrast to our previously published findings in lung tissue [21], we did not see a dependence of the phase relationship on the intrinsic period (τ *Before*) (Fig 2D). This discrepancy may either reflect tissue-specificity, or a fundamental design difference between single- versus multi-oscillator systems, potentially characterized by different degrees of cellular communication and, hence, different circadian network dynamics. Noguchi *et al*. [23] showed that paracrine signals from adjacent cells are necessary for the expression of robust fibroblast circadian rhythms on the population level. This indicates that intercellular communication is required for a coherent rhythmic output, even in cell cultures. However, the observed difference in circadian function between cell cultures (present study) and tissues (previous publications) suggests that the strength/impact of intercellular communication may be qualitatively different on a 2-dimensional (cultures) versus 3-dimensional (tissues) scale.

### Biphasic circadian phase responses to temperature are cell autonomous

The circadian phase response of single cells towards temperature events (Fig 3B) resembles those of multi-oscillators systems: in principle, the shape of the resulting response curve is in line with mammalian behavioral phase responses to light [31], with behavioral and electrical responses of mammalian tissues treated with chemicals mimicking light activation [32;33], and with light-responses of PER2::LUC-driven bioluminescence in photoentrainable fibroblasts [34;35]: a large portion of the curve displays delay shifts of up to 9 hours, and there is a smaller, less pronounced portion with advance phase shifts of up to 4 hours. It is noteworthy, however, that our response curve consists of a pronounced delay and a small advance portion, while many PRCs to non-photic stimuli have large advance portions only [36]. One possible explanation might be that we measured single cells, while non-photic PRCs are typically generated from organisms or organotypic tissues with the added benefit of potential coupling. Nevertheless, our biphasic curve to temperature stimuli is consistent with another PRC depicting bioluminescence shifts in response to 12h-temperature pulses in rat primary fibroblast cultures [19]. A typical PRC has a so-called “dead zone” of several circadian hours, where the system does not respond with any phase shift at all. This “dead zone” is not present in our data. However, this can be explained by the long duration of our temperature pulse: Comas *et al*. (2006) showed that the “dead zone” gradually disappears in PRCs with for stimulus durations larger than 6h. In conclusion, single cell phase responses are similar to multicell-level or even organismal responses, suggesting that the molecular ability to respond with delay or advance shifts in a time-dependent manner is cell-autonomous.

This seems to be different for the temperature-induced amplitude responses which did not reveal any time-of-day-dependent characteristics in our hands. Pulivarthy *et al*. [34] demonstrated phase-dependent amplitude changes in response to a light pulse in single photosensitive fibroblasts, while Ukai *et al*. [35], who used a similar photosensitive fibroblast system, did not find consistent single cell amplitude changes in response to a light pulse. Signal and recipient in the two studies mentioned above are very different from ours, rendering a direct comparison difficult. However, it demonstrates that different signal intensities might produce varying results within a similar system. Consequently, we cannot exclude that a stronger temperature stimulus would have resulted in cell-autonomous time-dependent amplitude responses in our system as well.

### Intercellular communication increases robustness in single cells

In order to evaluate the role of intercellular communication for circadian synchronization to temperature cycles, we used an intensively studied system which is known to exhibit synaptic communication in dispersed cultures: the circadian pacemaker in the SCN [25;37–40]. Unlike most studies that employed tissue slices and pharmacological treatments to investigate the impact of temperature on the SCN [12;16;41], we dispersed the pacemaker cells and seeded them at different densities, allowing for different rates of cellular communication and, perhaps, for different degrees of coupling. Our aim was to leave the pacemaker cells pharmacologically and genetically unperturbed. Compromising intercellular communication by culturing cells at lower density clearly reduced magnitude of bioluminescence, precision and smoothness in single cell traces (Fig 4A). Although cells cultured at medium density never exhibited rhythms that could be classified as “entrained” with the temperature cycle, single cells in a medium dense culture (i.e. presumably reduced cellular communication) definitely exhibit increased responsiveness to temperature. This is clearly illustrated in Fig 4B, where representative examples show an unperturbed free-running circadian rhythm in dense cultures, whereas rhythmicity in medium dense cultures adjusts itself according to the external temperature cycle. It is also reflected in circadian phasing (Fig 4C), which shifts towards peaking in the warm phase in medium dense cultures. Altogether, these findings illustrate a critical role for intercellular communication in the generation of robust circadian rhythmicity.

Increased entrainability to external stimuli has traditionally been linked to reduced rhythm amplitudes on the population level [12;16;22]. Our data show that it also correlates with amplitude reduction in single cells (Fig 4D). Consequently, this single cell amplitude reduction might be the key explanation for increased responsiveness to temperature in medium dense cultures. Whether it is caused by a lack of paracrine signals from adjacent cells, like in fibroblasts [23], or by reduced cellular coupling, remains to be determined. Our data are in agreement with Buhr *et al*. (2010), who showed that single cell rhythms in pharmacologically decoupled organotypic SCN slices (here: TTX-mediated sodium channel inhibition) display reduced relative amplitudes. On the other hand, single neurons from pharmacologically decoupled organotypic SCN slices (here: cAMP-inhibition) displayed unaltered relative rhythm amplitudes [12]. This apparent discrepancy can be explained by the different pharmacological strategies used to achieve decoupling: cAMP-inhibition interferes with VIP-mediated coupling, but–as far as we know—leaves the cell-autonomous molecular clock unperturbed, while TTX-mediated sodium channel inhibition, interferes with an important electrical feedback on single cell clock outputs [42]. In other words, TTX-mediated sodium channel inhibition probably impacts different parts of the circadian network compared to cAMP-inhibition, and, hence, the controversial results. Differential findings in physical (density-related, present results) and pharmacological decoupling [12] can be similarly explained. They indicate a strong impact of the mechanistic type of decoupling (dispersing vs. pharmacology). Conversely, this is probably also true for the type of coupling.

In summary, this is the first quantitative study of peripheral and central circadian single cell responses to periodic external stimuli. We demonstrated that single peripheral oscillators share many circadian traits common to multi-oscillator systems, like tissues or even organisms, but that there are also marked differences stressing the importance of intercellular communication for circadian function. Our findings show that the degree/quality of intercellular communication can vary significantly between tissues and emphasize the need for comparative studies, evaluating the degree/importance of network dynamics in a given tissue, before it may be a successful target for meaningful circadian manipulation.

## Supporting information

S1 Movie PER2::LUC bioluminescence of mouse primary fibroblasts exposed to a T20-temperature cycle with deltaT = 4K. The recording time in days is depicted in the top right corner. Culture temperature is shown in the bottom right corner. PER2::LUC fibroblasts were mixed with C57Bl/6 WT fibroblasts at a ratio of about 1:20, and cultures were grown to confluence before imaging. Upon imaging the serum concentration of the culture medium was reduced to 3% in order to prevent/reduce cell proliferation and mobility.
(MP4)

S2 Movie PER2::LUC bioluminescence of mouse primary fibroblasts exposed to a T24-temperature cycle with deltaT = 4K. The recording time in days is depicted in the top right corner. Culture temperature is shown in the bottom right corner. PER2::LUC fibroblasts were mixed with C57Bl/6 WT fibroblasts at a ratio of about 1:20, and cultures were grown to confluence before imaging. Upon imaging the serum concentration of the culture medium was reduced to 3% in order to prevent/reduce cell proliferation and mobility.
(MP4)

S3 Movie PER2::LUC bioluminescence of a mouse suprachiasmatic nucleus dispersal culture exposed to a T20-temperature cycle with deltaT = 2K. The recording time in days is depicted in the top right corner. Culture temperature is shown in the bottom right corner.
(MP4)
